# High activity of the stress promoter contributes to susceptibility to stress in the tree shrew

**DOI:** 10.1038/srep24905

**Published:** 2016-04-29

**Authors:** Hui Fang, Yun-Jun Sun, Yan-Hong Lv, Rong-Jun Ni, Yu-Mian Shu, Xiu-Yu Feng, Yu Wang, Qing-Hong Shan, Ya-Nan Zu, Jiang-Ning Zhou

**Affiliations:** 1 CAS Key Laboratory of Brain Function and Disease, School of Life Science, University of Science and Technology of China, Huangshan Road 443, Hefei 230027, Anhui, China

## Abstract

Stress is increasingly present in everyday life in our fast-paced society and involved in the pathogenesis of many psychiatric diseases. Corticotrophin-releasing-hormone (CRH) plays a pivotal role in regulating the stress responses. The tree shrews are highly vulnerable to stress which makes them the promising animal models for studying stress responses. However, the mechanisms underlying their high stress-susceptibility remained unknown. Here we confirmed that cortisol was the dominate corticosteroid in tree shrew and was significantly increased after acute stress. Our study showed that the function of tree shrew CRH - hypothalamic-pituitary-adrenal (HPA) axis was nearly identical to human that contributed little to their hyper-responsiveness to stress. Using CRH transcriptional regulation analysis we discovered a peculiar active glucocorticoid receptor response element (aGRE) site within the tree shrew CRH promoter, which continued to recruit co-activators including SRC-1 (steroid receptor co-activator-1) to promote CRH transcription under basal or forskolin/dexamethasone treatment conditions. Basal CRH mRNA increased when the aGRE was knocked into the CRH promoter in human HeLa cells using CAS9/CRISPR. The aGRE functioned critically to form the “Stress promoter” that contributed to the higher CRH expression and susceptibility to stress. These findings implicated novel molecular bases of the stress-related diseases in specific populations.

Stress is a condition that disturbs the physiological and psychological homeostasis of living organisms^1^,^2^ and can affect the organism throughout its lifespan^3^. Chronic or severe stressful life events will cause many stress-related diseases including cardiovascular diseases^4^, acute respiratory distress syndrome^5^, peptic ulcer^6^, immune and neuropsychiatric diseases^7^.

Corticotrophin-releasing hormone (CRH) is a principle regulator of the hypothalamic-pituitary-adrenal (HPA) axis, which is the canonical pathway that regulates the physiological response to stress^8^. Stress signals are received by the cortex and are integrated into the hypothalamus, then specific neurons in the paraventricular nucleus (PVN) of the hypothalamus produce and release the CRH, which binds to its CRHR1 receptor and controls the secretion of adrenocorticotropin hormone (ACTH) by the pituitary gland and corticosteroids by the adrenal glands. Corticosteroids enter circulation and act on many organs to coordinate the responses of the brain and the body to stress. In the hypothalamus, corticosteroids bind to glucocorticoid receptors (GRs) and regulate CRH expression and the HPA axis through a negative feedback mechanism^9^. Once in the nucleus, GRs bind to glucocorticoid response elements (GREs) and regulate transcription of target genes. GR mediated trans-activation requires the positive (+) GREs that belong to a family of imperfect palindromes consisting of two inverted hexameric half-site motifs separated by three base pairs^10^. The CRH promoter contains two canonical negative GRE elements in human and glucocorticoids negatively regulate CRH production in the hypothalamus^11^ . In this work we had discovered a new positive regulation of glucocorticoids in the CRH promoter that up-regulated CRH expression in Tree shrew hypothalamus. CRH expression appears to be linked to the controls of various nuclear steroid receptors^12^ including the androgen receptor^13^, estrogen receptor^14^, and retinoid receptor^15^. According to the chronic stress hypothesis, excessive CRH release and over-activation of the HPA axis, as well as defects in the negative feedback loop, are crucial events in the etiology of depression.

The tree shrew (Tupaia belangeri) has much closer circadian rhythm to human compared with rodents. It has been employed to study a variety of neurological diseases, - in particular, psychosocial stresses^16^,^17^ to which it has shown high vulnerability. During periods of daily social stress, male tree shrews develop symptoms similar to those observed in depressed patients, including persistent hyperactivity of the HPA axis, disturbed sleep patterns and reduced motor activities. Moreover, chronic psychosocial stress can cause physiological responses in the male tree shrew, such as body weight loss, elevated corticosteroid levels, reduced testosterone and developed depression-like behaviors. The social stress responses can be reversed or ameliorated by treatment with antidepressants such as tianeptine^18^ and fluoxetine^19^. However, the mechanisms underlying the high susceptibility to stress of tree shrew remained unknown. Moreover, the long debate on the evolutionary phylogenetic position of the tree shrew^20^,^21^,^22^ and the small number of related studies had limited the applications of the tree threw model for studying human diseases.

In the present study, we aimed to characterize the tree shrew CRH gene and compare its key regulators of HPA axis with those of human. Special attentions were paid to the molecular mechanisms underlying their susceptibility to stress, especially the transcription of CRH gene in regulating the HPA axis and the stress responses. In this study, the CRH gene sequence of tree shrew was annotated which shows high similarity with human CRH. Binding affinities of CRH to its receptor and CRH-CRHR1 signaling transduction assays demonstrated that the function of tree shrew CRH was almost identical to human CRH. An aGRE site within the tsCRH promoter was identified which functioned as a key element that contributed to the higher CRH expression levels in the tree shrew when compared with that of human under basal conditions and after treatment with forskolin and/or dexamethasone. Using Cas9/CRISPR technology, we further determined that when the tree shrew aGRE site was added to the CRH promoter in human HeLa cells, increased CRH mRNA expression was observed. The corresponding element identified in human CRH promoter region is polymorphic and a registered (-811A-G) SNP may affect its GR binding. Our results highlight the critical role of the aGRE site in regulating CRH mRNA expression through GR binding which results in increased promoter activity and susceptibility to stress in the tree shrew.

## Results

### Cortisol levels increase in response to acute stress in the tree shrew

**Figure 1:**
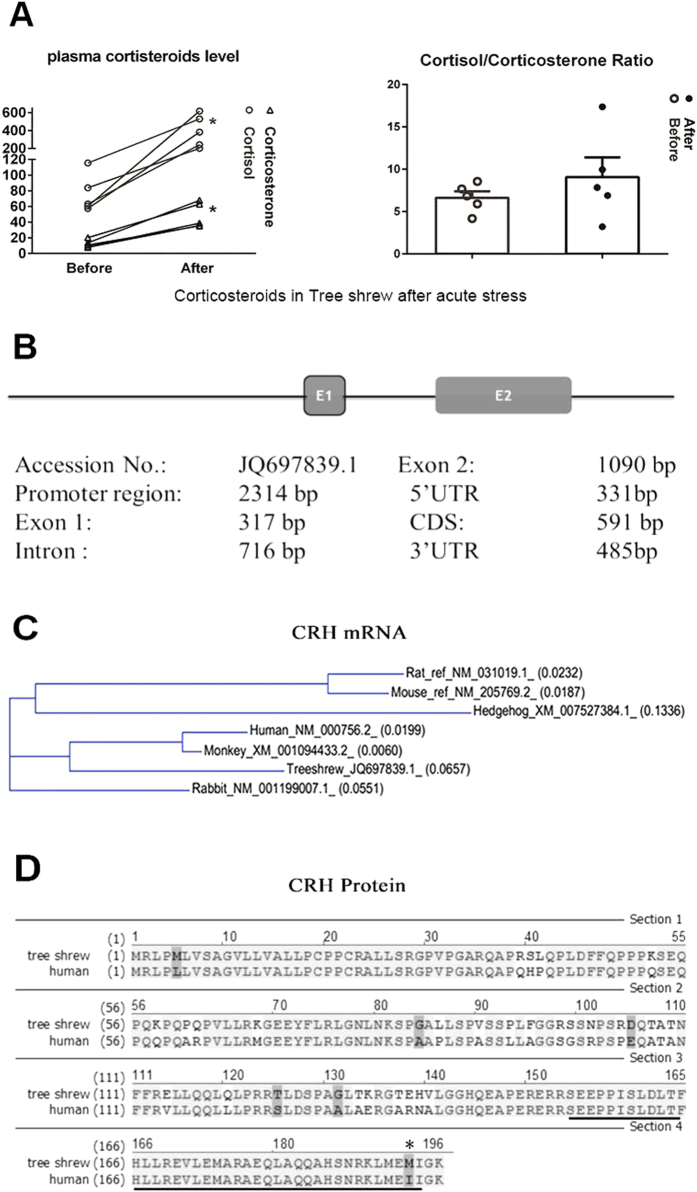
Acute stress in the tree shrew and analysis of its CRH gene. (**A** left) 15 min acute restraint stress on tree shrew had increased their mean plasma levels of cortisol and corticosterone from 76 ng ml^−1^ and 12.1 ng ml^−1^ (n = 5 between 1000 h and 1100 h) to 395.22 ± 80.31 ng ml^−1^, (cortisol) and 47.95 ± 7.14 ng ml^−1^ (corticosterone), but (A right) the ratio of cortisol/corticosterone were not influenced (*p < 0.05, paired t-test). (**B**) Tree shrew CRH genomic sequence was described in features including two exons separated by a 716 bp intron and encoded a 196 amino acid protein. (**C**) Phylogenetic tree analysis of resolved CRH mRNA alignments among human, monkey, tree shrew, hedgehog, rabbit, rat and mice. (**D**) Predicted protein sequence analysis showed a high similarity between human and tree shrew including an amino acid change (I40M, asterisk) in the secreted peptide domain (underlined).

Under normal conditions, the mean plasma levels of cortisol and corticosterone in the tree shrew were 76 ng ml^−1^ and 12.1 ng ml^−1^ (between 1000 h and 1100 h), respectively. After 15 min of acute restraint stress, the plasma levels of corticosteroids in the same tree shrew were significantly increased (cortisol, 395.22 ± 80.31 ng ml^−1^, p = 0.016; corticosterone, 47.95 ± 7.14 ng ml^−1^, p = 0.003) (Fig. 1A left; average increases of 500% and 400%, respectively), whereas the ratio of cortisol/corticosterone did not increase significantly (Fig. 1A right). Our data demonstrated that cortisol was the major corticosteroid involved in regulating the physiological responses to stress in the tree shrew, and high levels of cortisol is connected to stress susceptibility.

### Characterization of the tree shrew CRH gene

Approximately 5 kb of the tree shrew CRH gene sequence was resolved using the genome walking method (Fig. 1B). Through gene analysis and multiple sequence alignments, we observed a high degree of similarity of the tree shrew CRH gene compared with the human CRH gene corresponding to similarities of approximately 75% for the genomic DNA sequence and 85% for the mRNA sequence. We continued to perform a phylogenetic tree analysis of the mRNA sequences of a number of genes including CRH (Fig. 1C), CRHR1, CRHBP, POMC and GR (Supplementary Figure 1) which are the key mediators of HPA axis. The results revealed that there was a much shorter evolutionary distance between the tree shrew and primates than that of rodents (Fig. 1C and Supplementary Figure 1). The mature CRH peptides were processed from its precursor protein pro-CRH. Our sequence analysis revealed a single amino acid substitution (I40M) in the mature CRH peptide of the tree shrew (Fig. 1D).

**Figure 2:**
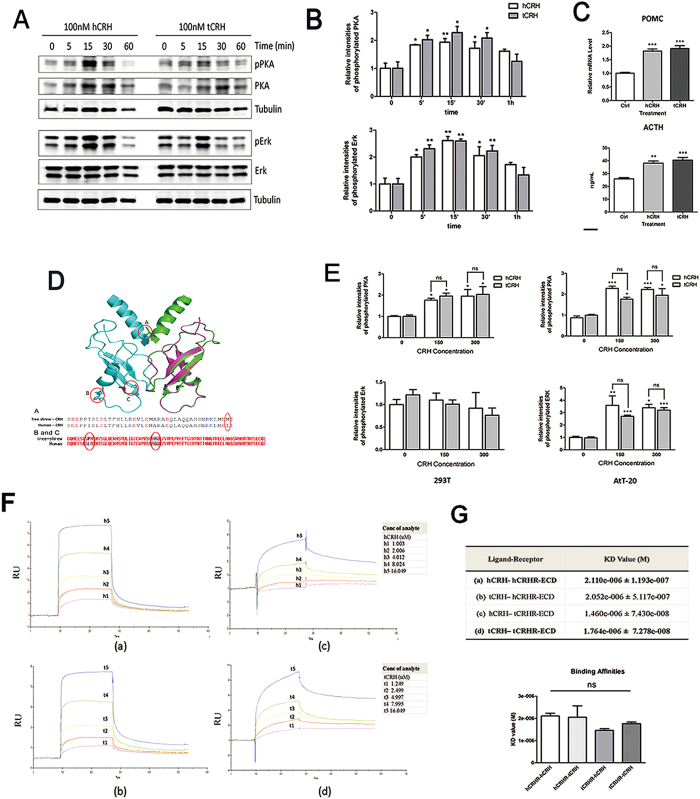
CRH peptides functioned similarly between human and tree shrew. (**A**) Activation of PKA and ERK signal pathways were induced by synthesized human (hCRH) or tree shrew CRH (tsCRH) peptides (10^−7^M) in AtT-20 cells. (**B**) Quantification of hCRH and tsCRH triggered signal pathway activation (*P < 0.05, **P < 0.01, n = 5, Two Way Anova). (**C**) Comparison of POMC transcription and ACTH releasing levels between the treatment of hCRH and tsCRH (10^−7^M) (**P < 0.01, ***P < 0.001, n = 5, One Way Anova). (**D**) Prediction of tree shrew CRH-CRHR1 binding complexes 3D structure according to the resolved human construction. Protein sequences alignments of (a) human and tree shrew CRH and (b,c) their CRHR1 receptors. The one amino acid mutation (I40M) of tree shrew CRH located at the C-terminal region for receptor binding. (**E**) Activation of PKA signal pathways in dose dependent way by hCRH and tsCRH in 293T and AtT-20 Cells over-expressing human or tree shrew CRHR1s (*P < 0.05, **P < 0.01, ***P < 0.001, n = 3, Two Way Anova). (**F**) Kinetic SPR binding curves of CRH peptides with immobilized CRHR1 extra-cellular domains and corresponding calculated Binding affinities (Kd). (**G**) Statistics of Kd values.

To evaluate the downstream effects of the single amino acid change, murine pituitary AtT20 cells were treated with synthesized human or tree shrew CRH peptides. Phosphorylation levels of PKA and ERK kinases were detected by western blot to evaluate the CRH induced signal pathway activations. Our results showed that both 100 nM human CRH (hCRH) and tree shrew CRH (tsCRH or tCRH) transiently activated ERK and PKA as early as at 5 min after treatment (pERK: hCRH p = 0.031, tsCRH p = 0.005; pPKA: hCRH p = 0.016, tsCRH p = 0.045) and reached peak activation at 15 min after treatment (pERK: hCRH p = 0.001, tsCRH p = 0.001; pPKA: hCRHp = 0.008, tsCRH p = 0.013; one-way ANOVA and Turkey), indicating a similar pattern of signal activation between hCRH and tsCRH (Fig. 2A,B). As cAMP/PKA and the ERK signaling pathways functions in controlling the expression of the CREB transcription factor and the Nur77 orphan receptor, which modulate POMC transcription in corticotrophs^23^, the hCRH and tsCRH induced increasing of POMC mRNA transcription and ACTH- the final POMC product-releasing levels, were examined in AtT-20 cells using real time Q-PCR and ELISA. Both hCRH and tsCRH incubation significantly up-regulated POMC mRNA transcription levels (hCRH p < 0.001, tsCRH p < 0.001) and increased the secretion of ACTH (hCRH p = 0.001, tsCRH p < 0.001; one-way ANOVA and Turkey). No significant differences were observed between hCRH and tsCRH treatment (pERK: p = 0.8164; pPKA: p = 0.3567; POMC: p = 0.703; ACTH: p = 0.394) (Fig. 2C).

### Tree shrew and human CRH demonstrated similar binding affinities to their receptor CRHR1 and similar capabilities to activate downstream signaling pathways

To determine whether the I40M substitution in tree shrew CRH would affect its protein structure and binding affinity to the receptor tsCRHR1, the secondary structures of dissolved hCRH and tsCRH were characterized using circular dichroism (CD) spectroscopy, and bioinformatical pre-analysis was used to predict the crystal structure of the tree shrew CRH-CRHR1-ECD (CRH and the Extracellular domain of CRHR1) binding complex based on the published human CRH-CRHR1-ECD structure. Our results revealed a refined conformational change in the secondary structures between tree shrew and human CRH, where tree shrew CRH contained a little lower α-helix and higher β-sheet contents (Supplementary Figure 2). Furthermore, crystal structure prediction revealed slight integral 3D crystal structure alterations of the tree shrew CRH and its ligand- CRHR1 extracellular domain binding complex compared to that of human (Fig. 2D). To further investigate whether the minor structure modifications of tree shrew CRH and its CRHR1 receptor could result in higher efficiency of CRH and CRH receptor-mediated signaling pathway activation compared to human, tsCRH and hCRH were added to AtT-20 and 293T cells over-expressing hCRHR1 or tsCRHR1 at two time points (5 min and 15 min) and doses (150 nM and 300 nM) (Fig. 2E). The results showed that both CRH peptides were capable of activating the cAMP/PKA pathway through their receptors to a similar degree in a time- and dose- dependent way, revealing that the modestly higher binding affinity between tsCRH and its receptor did not translate into a significant increase in downstream signaling activation (p = 0.8959 two-way ANOVA) (Fig. 2E).

We further identified the explicit binding affinities (Kd) of the human and tree shrew CRH ligands to their CRHR1 receptors respectively using surface plasma resonance (SPR), of which the specific binding kinetics were presented by RU curves recorded at 5 concentration gradients of hCRH and tsCRH (Fig. 2F). The results showed that among all the Ligand-receptor pairings, tsCRH binding to tsCRHR1 displayed relatively lower Kd value (176.44 nM ± 7.27 nM) than that of hCRH binding to hCRHR1 (Kd value was 211nM ± 11.93 nM). As lower Kd values indicate higher binding affinities, amino acid alterations in tree shrew CRH and CRHR1 protein sequences contribute to the mildly but not significantly increased binding affinity of the tsCRH-tsCRHR1-ECD complex compared to that of human (p = 0.799 one-way ANOVA and Turkey) (Fig. 2G).

### Higher transcriptional levels are driven by the three shrew CRH promoter

**Figure 3:**
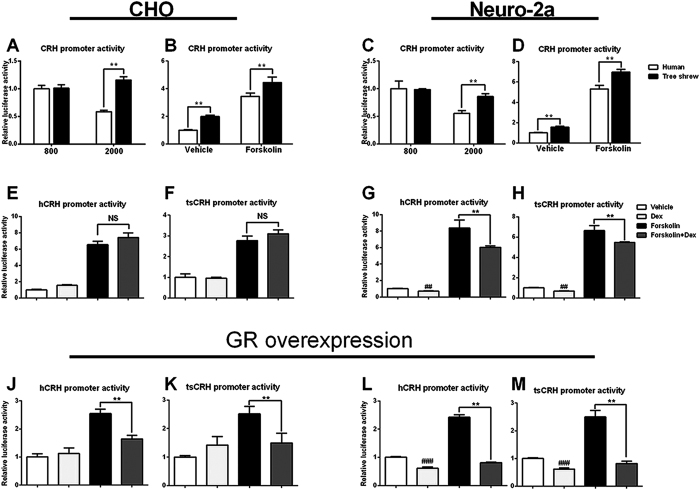
CRH promoter activity was conservatively regulated by CREB and glucocorticoid receptor. (**A**–**D**) CHO and Neuro-2a cells were transfected with luciferase vectors inserted with 800 bp or 2000 bp fragments from tree shrew or human CRH promoter region. Cells were treated with forskolin (10^−5^M) for 12 h before subjected to dual luciferase reporter assay. CRH promoter activity was much higher of 2 kb, not 800 bp tree shrew fragments than of human under native condition or treatment with forskolin (**P < 0.01, n = 3, Two way Anova). (**E–M**) CRH promoter activity was significantly enhanced under treatment with Forskolin (10^−5^M) and GR was required for Dexamethasone (10^−5^M) in counteracting the effect, under the overexpression of GR (**J**–**M**) or not (**E**–**H**) in CHO (**E**,**F**,**J**,**K**) and Neuro-2a (**G**,**H**,**L**,**M**) cells (**P < 0.01, ^**##**^P < 0.01, ^**###**^P < 0.001, n = 3, One Way Anova).

We simultaneously evaluated differences in transcriptional regulation of the CRH gene between tree shrew and human. To analyze CRH promoter activity, fragments encompassing the CRH promoter region (800 bp and 2000 bp in length) from the human and tree shrew genomes were sub-cloned into the pGL3-basic vector for a luciferase reporter assay. In both CHO and Neuro-2a cells, higher transcriptional activity for the 2-kb fragment but not the 800-bp fragment was observed in tree shrew CRH construct when compared with the human construct (Fig. 3A,C). These results suggest that cis-elements located within the −800 to −2000 bp region of the tree shrew CRH promoter may be responsible for the difference between the human and tree shrew promoters. Thus the 2-kb CRH promoter fragments were used in subsequent experiments to identify the key elements responsible for higher CRH transcriptional activity in the tree shrew. Because the transcription factors CREB^24^ and GR^25^,^26^ are involved in the regulation of CRH gene expression, we first determined the activity of the promoters in response to CRH transcription activation by forskolin-induced CREB activation and CRH transcription inhibition by dexamethasone-induced GR activation. When treated with forskolin, the 2 kb tsCRH promoter fragment consistently showed increased activity when compared with the human fragment in CHO (Fig. 3B) and Neuro-2a cells (Fig. 3D).

Low GR expression occurs in Neuro-2a cells and much lower GR expression occurs in CHO cells (Supplementary Figure 3). In both cell lines, forskolin enhanced CRH promoter activity in human and tree shrew constructs. If GR was not co-transfected into the cells, then forskolin combined dexamethasone (Dex) treatment decreased CRH promoter activity in Neuro-2a cells (Fig. 3G,H) but not in CHO cells (Fig. 3E,F). When GR was over-expressed in CHO cells (Fig. 3J,K) and Neuro-2a cells (Fig. 3L,M), the activities of the CRH promoter constructs were both significantly decreased in response to forskolin combined dexamethasone treatment. These results demonstrated that transcriptional control of the CRH gene by CREB and GR is conserved in the tree shrew.

### An active GRE site contributes to higher CRH promoter activity in the tree shrew

**Figure 4:**
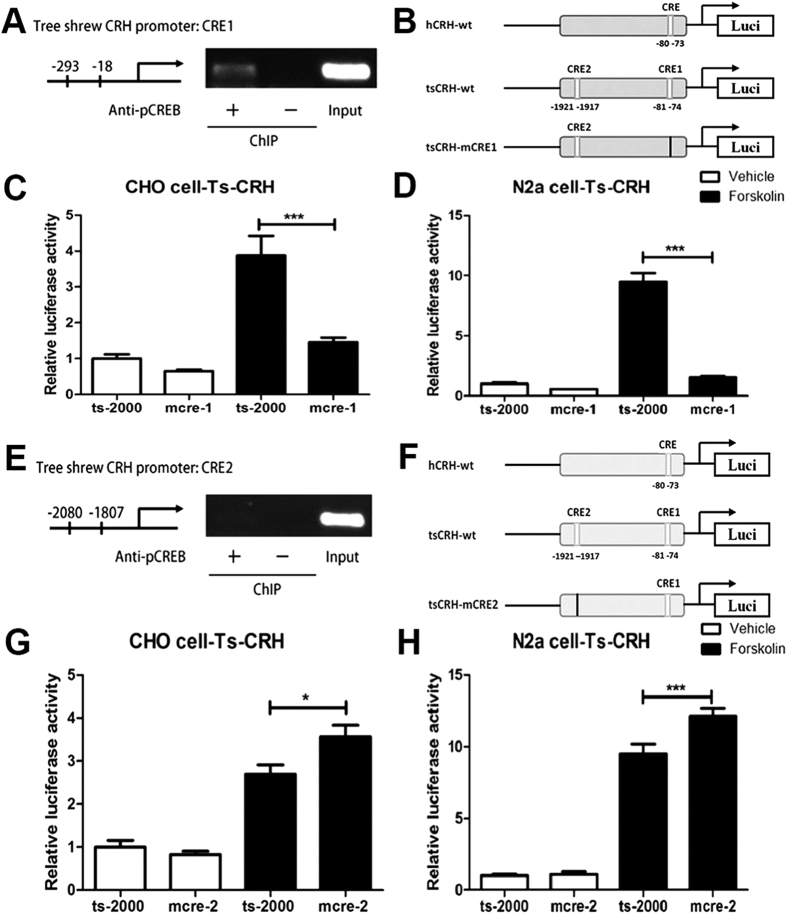
Higher promoter activity was not attributed to the predicted CRE sites in CRH promoter region of tree shrews. Transcription factor binding site prediction identified two CRE sites were located in the promoter region of CRH gene in tree shrews and only the proximal site (−81 to −74, **A**) but the distal site (−1921 to −1917, **E**) was confirmed functional by ChIP assay using pCREB antibody followed by PCR of the supposed tree shrew CRE sequences. In contrast to the decrease of promoter activity led by deletion of the proximal site (**B**–**D**), deletion of the distal site led to a little increase (**F**–**H**). (*P < 0.05, ***P < 0.001, n = 3, One Way Anova).

To identify the key cis elements responsible for higher promoter activity in the tree shrew, a transcription factor binding site search was performed using the TESS database. This analysis revealed a novel predicted CRE binding site in the tree shrew CRH gene promoter in addition to a corresponding proximal CRE site, which is not present in the human CRH gene promoter (Fig. 4B,F). Surprisingly, the results of ChIP assay using pCREB antibody showed only the fragment encompassing the proximal CREB binding site (−81 to −74, corresponding to −80 to −73 in the human CRH gene) was amplified (Fig. 4A), as the distal predicted site (−1921 to −1917) does not appear to be an actual CREB binding site (Fig. 4E). By deleting each predicted CREB binding sites (Fig. 4B,F) and performing a promoter activity analysis, we found that deletion of the proximal site significantly decreased the promoter activity in a luciferase reporter assay under basal conditions and following treatment with forskolin (Fig. 4C,D). Conversely, CRH promoter activity was increased after deletion of the distal CRE site, indicating that the distal site may be bound by a repressive transcription factor (Fig. 4G,H) and was not a real CREB binding site.

**Figure 5:**
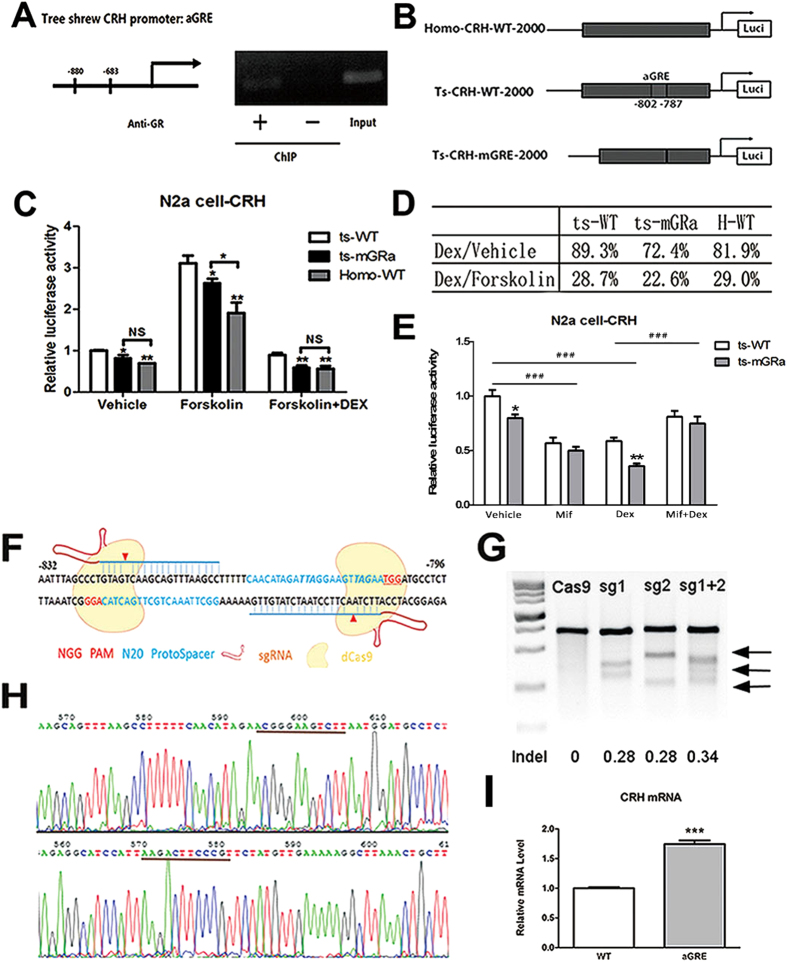
An active GRE site was responsible for higher CRH promoter activity in tree shrews. (**A**,**B**) TF binding site prediction analysis revealed an active GRE (aGRE) site was located in the CRH promoter region of tree shrew and it was confirmed by ChIP assay using GR antibody and PCR of tree shrew aGRE. (**C**) Promoter activity analysis revealed that the difference of CRH promoter activity between human and tree shrew diminished along with the depletion of the aGRE site (*P < 0.05, **P < 0.01, n = 3, Two Way Anova). (**D**) Statistical table showed the CRH promoter activity of mutant tree shrew retained a lower activity than wild type after treatment with dexamethasone. (**E**) The aGRE mutation caused a significant decrease of luciferase activity compared to wild type under the treatment of dexamethasone or control solvent. Mifepristone + dexamethasone treatment significantly antagonized the inhibition effect of dexamethasone on CRH promoter activities in N2a cells transfected with wild type or aGRE mutation reporter plasmids. (*P < 0.05, **P < 0.01, ^**###**^P < 0.001, n = 4, Two Way Anova). (**F**–**I**) CRH mRNA expression was increased after the aGRE site (underlined) was introduced into the CRH gene locus by a pair of guide RNAs (blue) using Cas9/CRISPR tech (*P < 0.05, **P < 0.01, n = 3, One Way Anova).

Transcription factor binding site prediction analysis also revealed an active glucocorticoid receptor binding site (aGRE) located in the tsCRH promoter region (−802 to −787: AGAACG GGA AGTCTTCT). It is highly consistent with the canonical + GRE consensus sequence AGAACA NNN TGTCCG in which the underlined G and C are the most essential bases^27^. The corresponding potential aGRE site identified in human CRH promoter (hGRE −824 to −808: AGATTA GGA AGTTAGAA) is an atypical element with impaired function, where a SNP rs192322083 (A–G) was found that may affect its binding ability to GR. ChIP assay using an monoclonal GR antibody followed by PCR confirmed that the predicted site we found is capable to bind GR (Fig. 5A). In Neuro-2a cells, after the loss-of-function mutation of deleting the aGRE site (Fig. 5B), tree shrew CRH promoter activity was significantly decreased in the presence or absence of forskolin and forskolin + dexamethasone treatment to a level that was similar to the hCRH promoter (Fig. 5C). After treatment with dexamethasone, the mutant tree shrew CRH promoter exhibited lower activity than the wild-type promoter (Fig. 5D; 72.4% vs. 89.3% in untreated cells, and 22.6% vs. 28.7% in forskolin-treated cells). To find out how GR antagonist affect aGRE activity, we used mifepristone alone or combined with dexamethasone to treat N2a cells transfected with CRH promoter reporter plasmids (Fig. 5E). When applied with dexamethasone or control solvent, the aGRE mutation caused a significant decrease of luciferase activity comparing with wild type, indicating the key role of the aGRE that contributed to high CRH promoter activity. Like dexamethasone, mifepristone treatment alone significantly decreased CRH promoter activity by acting as GR agonist partly, which is consistent with previous reports^28^,^29^. Compared with dexamethasone treatment, the combined use of mifepristone significantly antagonized the inhibition effects of dexamethasone on promoter activities of wild type and aGRE mutation. Meanwhile, dexamethasone showed a stronger inhibition effect on the CRH promoter of aGRE mutation than wild type (55% vs 41%). Conversely, mifepristone had a stronger antagonism effect on dexamethasone induced inhibition of CRH promoter activity in the aGRE mutation group (206% vs 139%). Next, we generated a knock-in cell line in which the active GRE site was introduced into the CRH gene promoter locus by Cas9/CRISPR technology^30^,^31^. A web-based design tool was used to design two guide RNAs (gRNAs) to target the locus (Fig. 5F), and Cas9 was transfected along with the gRNA or an empty vector into 293T cells. DNA electrophoresis confirmed that two of the designed gRNAs were capable of inducing double strand breaks at the expected site in 293T cells with approximately 30% in-del efficiency (Fig. 5G). We cloned the amplicon from the two gRNAs in 293T cells into a homemade T vector and identified 4 mutations out of the 16 randomly sequenced clones (Supplementary Fig. 4). To introduce the aGRE site into the CRH gene locus, human-derived epithelial carcinoma HeLa cells were transfected with the pair of gRNAs and Cas9 nickase (D10A) along with donor DNA. DNA sequencing revealed that the GRE site was successfully introduced into the CRH gene locus (Fig. 5H). In this knock-in clone, the basal level of CRH mRNA transcription was increased (p = 0.0003, two-tailed t-test) (Fig. 5I).

### GR recruits transcription factors to the active GRE site to form a trans-activation complex to positively regulate CRH gene expression

**Figure 6:**
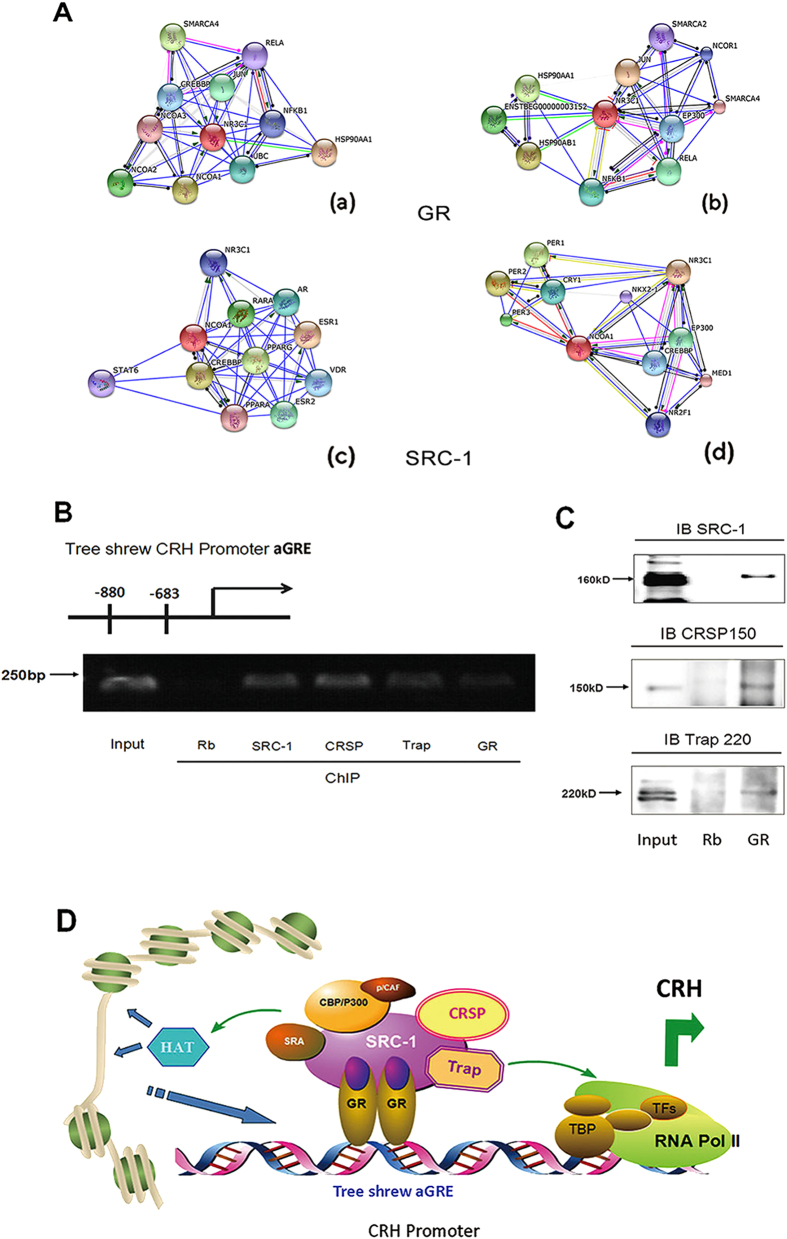
The active GRE site recruits trans-activators to form complexes for positive modulation of CRH transcription in Tree shrew. (**A**) STRING analysis of protein interaction networks of GR and SRC-1 related transcription factors in human and tree shrew. (a,c) Human, (b,d) Tree shrew. (**B**) ChIP Assay of co-activators associated with aGRE bound GR dimers were performed using antibodies against SRC-1, CRSP150 and Trap220 in tree shrew cortex and hypothalamus brain tissues. (PCR primers targeted at the aGRE sequence) Those factors form complex with GR to bind the aGRE site. (**C**) Co-IP Assay of GR interaction with co-activators. IP Antibody: GR, IB Antibody: SRC-1, CRSP150 and Trap220. (**D**)Schematic of GR organized activation complex in initiating CRH transcription.

To function as a nuclear transcription factor, activated GR recruits various co-activators to remodel the chromatin structure and to form and maintain a transcriptional pre-initiation complex at the aGRE site of the CRH gene promoter region^32^. Using the STRING web database we studied the protein interaction networks of GR (NR3C1) and transcription co-activator SRC - 1(NCOA1) related co-factors in Human and Tree shrew (Fig. 6A). Intimate connection of GR and SRC-1 in human was verified (Fig. 6A: a and c) while the evidences was limited in tree shrew (Fig. 6A: b and d). To determine whether the aGRE site interacts with trans-activation factors, a ChIP assay was conducted in brain tissues obtained from the tree shrew cortex and hypothalamus using antibodies against GR, SRC-1, CRSP150 (subunit of co-factor required for Sp1 activation) and Trap220 (subunit of the thyroid hormone receptor-associated protein complex). These factors participate in aGRE-mediated CRH gene transcription activation by stress-related factors (Fig. 6B). A co-IP assay using GR antibody in tree shrew tissues further confirmed that these co-activators interacted with each other forming a trans-activation complex (Fig. 6C). In the context of previous findings, we hypothesize that the aGRE-mediated CRH transcription from the tree shrew promoter is initiated by DNA-bound GR dimers that bind with the co-activator SRC-1. This complex subsequently recruits CBP, a CREB binding protein, to facilitate chromatin remodeling by the acetylation of histones^32^. Simultaneously, GR and SRC-1 recruit the factors TRAP and CRSP to form the activation complex, which stabilizes the pre-initiation complex and enhances mRNA synthesis by polymerase II (Fig. 6D).

## Discussion

The taxonomic history of tree shrews are complicated and had raised long debates^22^,^33^,^34^,^35^. As the tree shrew brains possess many primate characteristics^36^, they had been considered to have close affinities with primates and are often used as an out-group in analyses of relationships among primate taxa, whereas researches including analysis of mitochondria genes^37^ as well as Maximum likelihood and Bayesian analysis of 26 genes^34^ suggested that tree shrews have closer affinities to rodents than primates. Ultimately they were classified in the order Scandentia^38^,^39^. Our study suggested they have high similarities to primates in terms of the HPA-axis-mediated stress response regulation. Recent genomic study also supported the rationality of using tree shrew as an adjunct animal model to primates^22^. The “Year of the tree shrews” starts from 2000, heralding major advances in their genetics, behavioral ecology, and morphology^39^, as well as largely emerging applications of them in Pathological and Pharmacological studies^22^ .

Corticosteroids are important mediators of stress responses and are shaped by both genetics and external factors early in life^4^,^40^. In this study, our data suggest that plasma levels of both cortisol and corticosterone increase significantly in the tree shrew after acute restraint stress, which is consistent with previous data reported for primates and rodents that subjected to stress^41^,^42^. In tree shrews, blood cortisol circulates at levels that are 10- to 20- fold higher than corticosterone, which is similar to the ratio observed in human plasma^43^,^44^. Consistent with these observations in primates, cortisol is the principal glucocorticoid in the tree shrew, while in rodents corticosterone is the predominant one. Besides, our characterization of the CRH gene and the CRH signal pathway of the tree shrew also supported their high similarities to primates on the HPA axis and behavioral adaptations in response to stress. Researches have indicated the relative levels of expression and distributions of key mediators of stress responses differ between rodents and catarrhine primates^11^,^45^.

We determined the sequence of the tree shrew CRH gene and identified an amino acid substitution in the mature CRH peptide relative to human CRH, next we found that tree shrew and human CRH induced similar responses through their respective HPA axis activation. We simultaneously investigated the transcriptional regulation of the CRH gene. After confirming that tree shrew CRH gene activity is controlled by canonical CREB and GR signaling, we identified an aGRE site in the CRH promoter region functioning as a key cis element, which contributes to the increased CRH transcription activity that lead to the high vulnerability of the tree shrew to stress. These observations updated the previous view that CRH is negatively regulated by glucocorticoids through the function of GR as a trans-repressor that bind to the CRH promoter in the hypothalamus^26^,^46^, meanwhile they agreed with previously reported positive roles of GR on CRH in the placenta^47^. When the mutations of deleting key functional bases were introduced into the aGRE (mGRE), the CRH promoter activities were significantly decreased in all of con, foskolin and foskolin/dexamethasone treatment groups compared to wild type. The mutant tree shrew mGRE retained a higher promoter activity than wild type hGRE after treatment with forskolin indicated the possible underlying mechanisms of the interactions between the aGRE with CRE in regulating CRH production during stress in tree shrew, as previously reported that the mutual antagonism between CRE-like and composite GRE sites could serve as a mechanism by which glucocorticoids rapidly suppress cAMP and noradrenaline-stimulated TRH transcription^48^. Mechanistic studies have shown that GR transactivation requires: the presence of (+) GREs, which allosterically mediate GR binding and have consensus sequence with the aGRE, as well as recruitment of co-activators and transcription initiation^27^. When binding with glucocorticoids, GR activates or represses gene transcription by binding directly to active GR response elements (aGRE) or negative GR elements (nGRE) in association with co-activators or co-repressors^10^. In this study, we confirmed that GR binds to the aGRE site in association with SRC-1 in the tree shrew CRH promoter. These observations are consistent with previous study which suggested that SRC-1 was involved in the regulation of the CRH gene^49^. The tree shrew aGRE function in the recruitment of trans-activators such as CRSP and Trap by combining with SRC-1 that form co-activation complexes such as DRIP families that interact with multi-complexes^32^ in the CRH promoter through transcription factor synergism. Whether other factors participate in the high-stress susceptibility phenotype observed in the tree shrew remains to be further discovered.

Similar to our study on transcription regulation of CRH gene, A SNP (-248C-T) within the CRH gene that has been identified in rhesus macaques appears to contribute to increased CRH promoter activity, meanwhile a similar SNP (-201C-T) has been found in human although little is known regarding its function^45^. In humans, polymorphisms in the 5′ regulatory region of the CRH gene have been reported. Evidences of CRH promoter polymorphisms representing a genetic marker for susceptibility of rheumatoid arthritis (RA), a stress related immune disorder, in various ethnic groups of patients have been found^50^. Recently, a T-allele of SNPGR found in Japanese and Chinese populations have been confirmed to increase the activity of the TAC1 promoter through de-sequestration or de-repression of a highly conserved GR response sequence (2GR)^51^. The aGRE site discovered in the highly active tree shrew CRH promoter prompted us to consider a link between CRH promoter SNPs and individual susceptibility to stress-induced depressive disorders. Future case-control population studies focused on the gene of CRH and CRH promoter as well as the HPA axis will help uncover potential SNPs linked to stress susceptibility in human.

Furthermore, the *in vivo* studies to demonstrate the role of aGRE function such as how excess exogenous cortisol affects CRH mRNA expression in the hypothalamic PVN region under basal or stressed conditions remains to be investigated. Previous study of long-term chronic exogenous cortisol treatments on tree shrews showed that their urinary cortisol value was 6–9 times the basal level, while exogenous corticosterone administration on rats that raise plasma hormone to 4 mg/100 ml (basal levels up to 3 mg/100 ml) induced significant reduction in the diurnal corticosterone secretion^52^, which indicated that the HPA axis was hyper-activated in stress responses of tree shrew and their high susceptibility to stress. In a chronic psychosocial conflict study of tree shrews, stress significantly reduced the number of CRH binding sites in the anterior pituitary, as well as limbic and cortical structures of the brain, an effect consistent with the idea of receptor down-regulation due to elevated high concentrations of the endogenous ligand CRH^53^.

In conclusion, our findings provide a molecular basis to explain the high stress susceptibility of the tree shrew, a promising animal model for the study of the stress response and stress-related diseases.

## Materials and Methods

### Animals

Tree shrews were obtained from Kunming zoology institute, Chinese Academy of Science. All of the animal experiments were performed in accordance with the National Institute of Health Guide for the Care and Use of Laboratory Animals with approval from the Institutional Animal Care and Use Committee for University of Science and Technology of China.

### Genome walking and sequence description

Genome walking kit was purchased from Clontech Inc, (Mountain View, California) and performed according to the manufacturer’s instruction. Genomic DNA was extracted from the tree shrew kidney. The complete coding sequence of CRH gene was deposited into NCBI database (accession no.: JQ697839.1).The primers used to resolve full length CRH gene and used in the following ChIP assays were both listed in Table 1. GeneTrap and Vector NTI (Invitrogen, Carlsbad, California) was used in this study to analyze the gene sequence.

### SPR Analysis

Eukaryotic expressed hCRHR1-ECD and tsCRHR1-ECD proteins were provided by Cusabio (Wuhan, China). SPR assays were launched on the BIACORE3000 (GE Healthcare, Fairfield, Connecticut) apparatus. The 10 mM NaAc buffer (pH 4.5) was chosen to dilute receptor-ECDs from 1 mg ml^−1^ into 50 μg ml^−1^ and the proteins were immobilized on the sensor chip (GE) with around 2000 resonance unit (RU) increase. For kinetic measurement of interactions between CRH and CRHR1-ECDs, the protein samples were diluted in PBS buffer for SPR (0.137 M NaCl, 0.0027 M KCl, 0.0043 M Na_2_HPO_4_, 0.0014 M KH_2_PO_4_, pH7.4). Serially diluted ligands were injected at a flow rate of 30 μl min^−1^ for 3 min respectively, followed by a 2 min dissociation process per reaction. The response was detected as a function of time (sensorgram) at 25 °C.

### Cell culture, treatment and transfection

AtT-20, 293T, HeLa, Neuro-2a and CHO cells, purchased from ATCC, were grown in DMEM supplemented with 10% FBS and maintained in the 37 °C incubator containing 5% CO_2_. For CRH induced signal activation Assay, after treatment with hCRH and tsCRH peptides (China peptides, Shanghai, China) for different time courses (0, 5 min, 15 min, 30 min, 1 h) or doses (50 nM, 150 nM, 300 nM), cells were lysed in RIPA Buffer (Sigma, Santa Clara, California) containing phosphatase and protease inhibitor cocktails (Pierce, Thermo, grand island, New York), samples were collected in loading buffer (Bio-Rad, Hercules, California) and heated at 100 °C for 5 min. For transfection, 12 h after seeding, cells were co-transfected with reporter vector and a control plasmid phRL-TK (Promega, Madison, Wisconsin) using X-treme 9 DNA transfection reagent (Roche, Basel, Switzerland) according to the producer’s manual.

### Western blotting

Signal activations were detected by Western blot. Phosphorylation levels of p-PKA, p-Erk, PKA and Erk were immune-labeled by primary antibodies (Cell Signaling Technology, CST. Danvers, Massachusetts 1:1000) and HRP conjugated secondary antibodies (Promega). Signals were developed by chemiluminescence (West PICO Pierce) followed by capturing and quantifying by Lumi-imaging system (Bioshine ChemiQ Series, Shanghai, China).

### Real-time quantitative PCR of POMC transcription

Total RNA were isolated from AtT20 cells with 24 h of 100 nM hCRH and tsCRH incubation using Trizol Reagent (Sigma), and then were reverse transcribed and subjected to real time Q-PCR:

Primers (5′to 3′): Glyceraldehyde-3-phosphate dehydrogenase (GAPDH)

TCAACGGATTTGGTCGTAT (F), ATGAGTCCTTCCACGATAC (R);

Proopiomelanocortin (POMC):

GTGCCAGGACCTCACCAC (F), CTTCCGGGGGTTTTCAGT (R);

The reactions were performed in the ABI PRISM 7300 Detection System.

### Elisa of ACTH Releasing

ACTH levels in supernatants (diluted by ddH_2_O 1:5) from AtT-20 cells incubated with hCRH and tsCRH for 8 h were measured with ACTH Elisa Kits (Uscn, Wuhan, China).

### Constructs and mutagenesis

800 or 2000 bp fragments corresponding to CRH promoter region were amplified using genomic DNA as the template and ligated into the luciferase reporter vector pGL3-basic (Promega) between *KpnI* and *BglII* or *XhoI* sites. Mutation was introduced by using Quickchange mutation kit (Stratagene) according to the instruction.

### Promoter activity analysis

24 h after transfection, cells were collected and subjected to lysis in passive lysis buffer and luciferase activity was detected on Veritas Luminometer (Turner Biosystem, Atlanta, Georgia) using dual luciferase reporter assay (Promega), in which *Renilla* luciferase activity was used to normalize the transfection efficiency.

### Chromosome immunoprecipitation and Co-immunoprecipitation assay

For ChIP and Co-IP assays, male tree shrew was decapitated, and the cerebral cortexes and hypothalamus were obtained. Solutions containing soluble chromatins from tree shrew tissues were immunoprecipitated with the anti-GRα antibody, anti-pCREB antibody (CST) as well as anti-SRC-1, anti-CRSP150, anti-Trap220 antibodies (Santa Cruz, Dallas, Texas). Corresponding IgG proteins were used as negative controls. Subsequently DNA extraction, purification and amplification by PCR were performed with the primers listed in Table 1. The primers for ChIP assays are targeted to the promoter region of the CRH gene from −2080 to −1807 bp, −293 to −18 bp and −880 to −683 bp, respectively.

### Cas9/CRISPR mediated genome modification

293T cells were seeded 24 h before transfection. A Cas9+sgRNA (1:1 molar ratio) plasmid was transfected into cells using X-treme 9 DNA transfection reagent (Roche) at 50% confluency. 48–72 h later, cells were collected for genomic DNA extraction (Mag-MK genomic DNA extraction kit, Sangon, Shanghai, China) and PCR analysis. The genomic region flanking the CRISPR target site for CRH gene was PCR amplified, and products were purified using Gel DNA extraction kit (Axygen, Corning, New York). The purified PCR products were mixed with T7 endonuclease buffer (NEB, Ipswich, Massachusetts) and subjected to a re-annealing process. After re-annealing, products were treated with T7 endonuclease I and tested by DNA gel electrophoresis. Indel percentage was determined by the function:


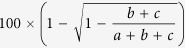


*a* stands for the integrated intensity of the undigested PCR band, and *b* and *c* are the integrated intensities of each cleavage band.

For Cas9 nickase mediate genomic modification, donor plasmid DNA was co-transfected with plasmid in total of Cas9 nickase and two sgRNAs into Hela cells. 24 h later, cells were screened with puromycin (5 ng ml^−1^) for 24–48 h, then were subcultured at very low density to enable cell clone formation. Each single clone was picked and cultured for 7 d, then subjected to genomic DNA and total RNA extraction and RT-qPCR analysis.

### Statistical analysis

Statistical analysis was performed using SPSS and Graph pad Prism 6.0 software. Significance of observations between data groups were established by paired or unpaired Student’s t test as well as analysis of variances followed by Turkey’s post hoc test. Analyzed data are indicated as mean ± s.e.m. The threshold of significance was set at P < 0.05, *, ** and *** represent P  <  0.05, P < 0.01 and P < 0.001, respectively.

## Additional Information

**How to cite this article**: Fang, H. *et al.* High activity of the stress promoter contributes to susceptibility to stress in the tree shrew. *Sci. Rep.* **6**, 24905; doi: 10.1038/srep24905 (2016).

## Supplementary Material

Supplementary Information

## Figures and Tables

**Table 1:** Statistical analysis of CRH promoter activity between human and tree shrew.

	ts-WT	ts-mGR	H-WT
Dex/Vehicle	89.3%	72.4%	81.9%
Dex/Forskolin	28.7%	22.6%	29.0%
