# Supplementary material for: High activity of the stress promoter contributes to susceptibility to stress in the tree shrew
Source: Sci Rep. 2016 Apr 29;6:24905. doi: 10.1038/srep24905 (PMC4850381; doi:10.1038/srep24905)
Supplement: Supplementary Information [file srep24905-s1.pdf]

High activity of the stress promoter contributes to susceptibility to stress in the tree shrew

Hui Fang*, Yun-Jun Sun*, Yan-Hong Lv, Rong-Jun Ni, Yu-Mian Shu, Xiu-Yu Feng, Yu Wang, Qing-Hong Shan, Ya-Nan Zu, Jiang-Ning Zhou

CAS Key Laboratory of Brain Function and Disease, School of Life Science, University of Science and Technology of China, Huangshan Road 443, Hefei 230027, Anhui, China

Corresponding author:

Jiang-Ning Zhou, M.D., Ph.D.

School of Life Science, University of Science and Technology of China,

Huangshan Road 443, Hefei 230027, Anhui, China;

Tel: 0086 551 63607658;

Fax: 0086 551 63607778;

E-mail: jnzhou@ustc.edu.cn

*These authors contributed equally to the work.

Running title: Stress Promoter in Tree Shrew

Author Information:

Hui Fang, Ph.D.   E-mail: fanghui@ustc.edu.cn;

Yun-Jun Sun, M.S.   E-mail: yjunsun@ustc.edu.cn;

Yan-Hong Lv, M.S.   E-mail: lvyanh1988@163.com;

Rong-Jun Ni, Ph.D.   E-mail: nrj2008@mail.ustc.edu.cn;

Yu-Mian Shu, M.S.   E-mail: soutine@mail.ustc.edu.cn;

Xiu-Yu Feng, M.S.   E-mail: fxyu@mail.ustc.edu.cn;

Yu Wang, M.S.   E-mail: wangyu10@mail.ustc.edu.cn;

Qing-Hong Shan, M.S.   E-mail: shanqh@mail.ustc.edu.cn;

Ya-Nan Zu, M.S.   E-mail: kknlyjqx@mail.ustc.edu.cn.

Supplementary Figure Legends

Supplementary Fig. 1 Phylogenetic tree analysis of CRHR1, CRHBP, POMC and GR mRNA alignments among human, monkey, tree shrew, hedgehog, rabbit, rat and mice.

Supplementary Fig 2 (A, B) Circular Dichroism Spectrum (CD) detection of human and tree shrew CRH (hCRH and tCRH) secondary structures in dissolved state. (C) Quantitative analysis showed that there were slight but not remarkable conformation changes in secondary structures of dissolved CRH peptides between tree shrew and human with the former had relative lower α-helix and higher β-sheet proportions.

Supplementary Fig 3 RT-PCR analysis of GR mRNA. Low GR expression in N2a cells and much lower expression in CHO cells were identified.

Supplementary Fig 4 Mutant 293T cell line in which aGRE site was introduced to CRH gene locus by Cas9/CRISPR technology. 4 mutations were found out of randomly sequenced 16 clones in the cloned amplicon in homemade T vector from 293T cells guided by two gRNAs targeting the anticipatory site.

Supplementary Figures:

Supplementary Fig. 1

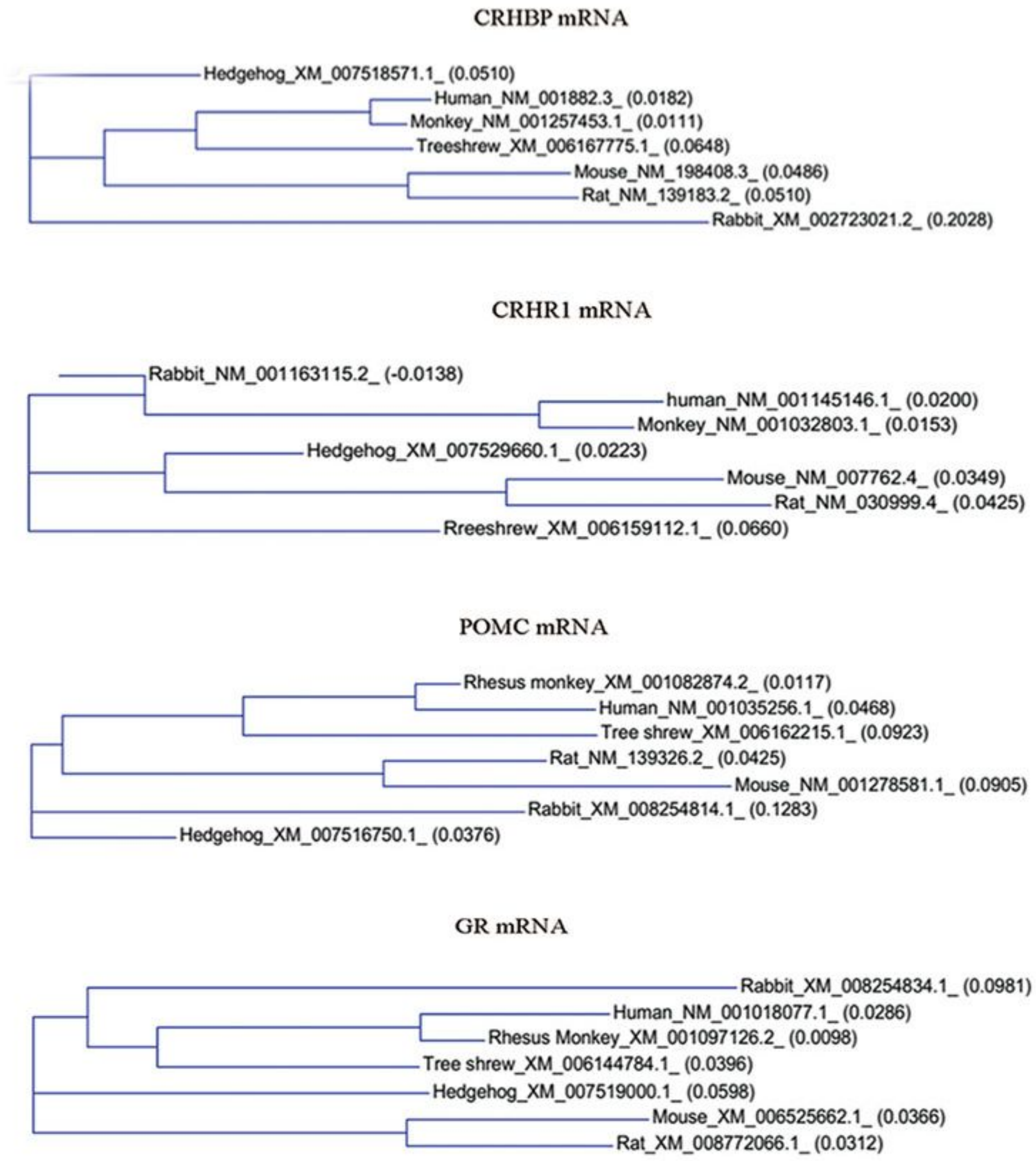

Supplementary Fig. 2

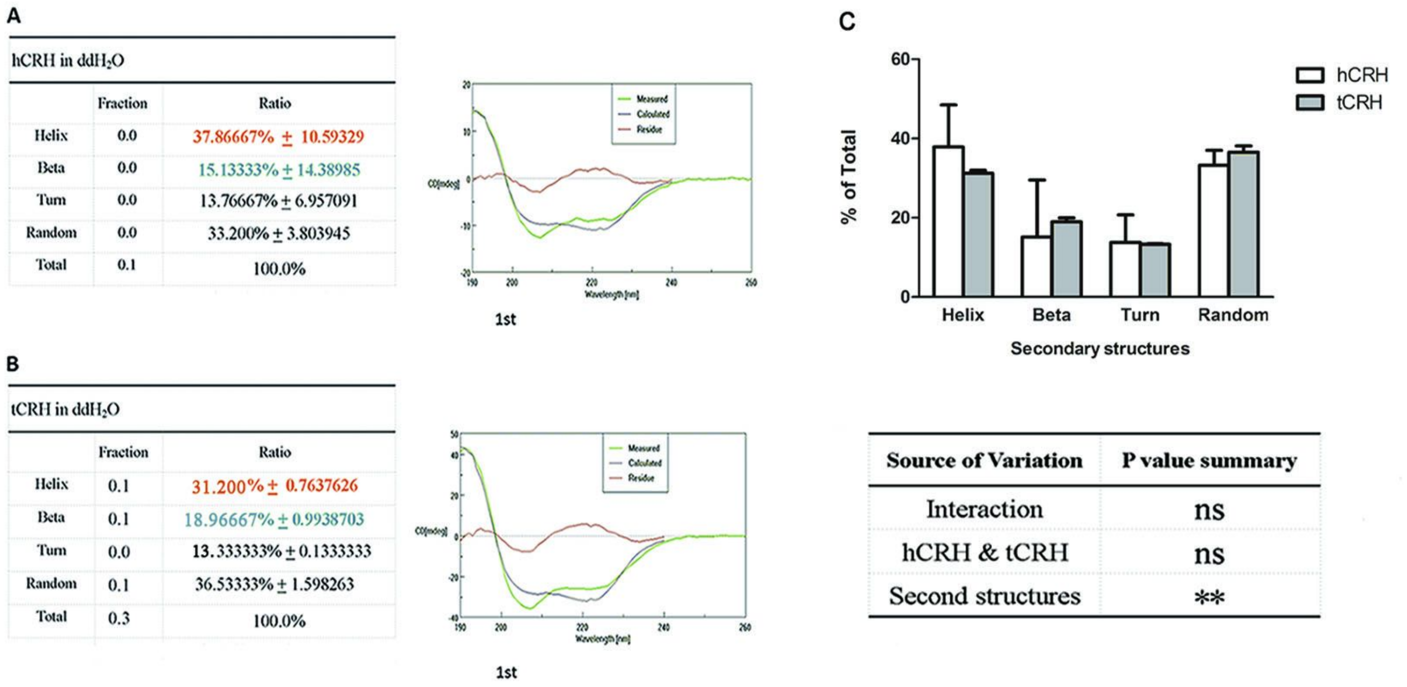

A hCRH in ddH_2O

	Fraction	Ratio
Helix	0.0	37.86667% ± 10.59329
Beta	0.0	15.13333% ± 14.38985
Turn	0.0	13.76667% ± 6.957091
Random	0.0	33.200% ± 3.803945
Total	0.1	100.0%

B tCRH in ddH_2O

	Fraction	Ratio
Helix	0.1	31.200% ± 0.7637626
Beta	0.1	18.96667% ± 0.9938703
Turn	0.0	13.333333% ± 0.1333333
Random	0.1	36.53333% ± 1.598263
Total	0.3	100.0%

Source of Variation	P value summary
Interaction	ns
hCRH & tCRH	ns
Second structures	**

Supplementary Fig. 3

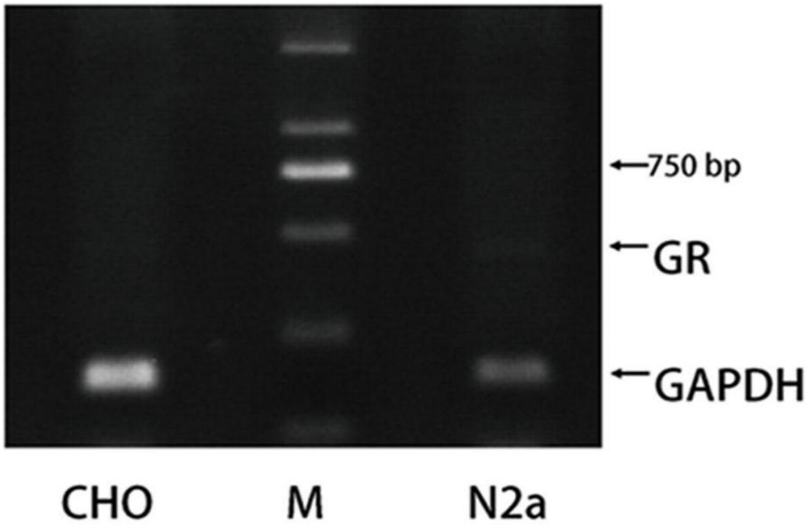

Supplementary Fig. 4

Mutations in 4 out of 16 sequenced alleles(sg1&sg2)

AATTTAGCCCTGTAGTCAAGCAGTTTAAGCCTTTTTCAACATAGATTAGGAAGTTAGAATGGATGCCTCTCATTGAGTTTGC wild type

AATTTAGCCCTGTAGTCAAGCAGTTTAAGCCTTTTTCAACATAGATTAGGA-------ATGGATGCCTCTCATTGAGTTTGC -7

AATTTAGCCCTGT---------------------TTCAACATAGATTAGGAAGTTAGAATGGATGCCTCTCATTGAGTTTGC -21

AATTTAGCCCTGTA------------------------ACATAGATTAGGAAGTTAGAATGGATGCCTCTCATTGAGTTTGC -24

AATTTAGCCCTGTAGTCAAGCAGTTTAAGCCTTTTTCAACATAGATTAGGAAGTTAaagcagtttaagccttttttcaacatagattaggaagttaGAATGG +36 (-4,+40)
